# Time to Development of Anemia and Predictors among HIV-Infected Patients Initiating ART at Felege Hiwot Referral Hospital, Northwest Ethiopia: A Retrospective Follow-Up Study

**DOI:** 10.1155/2020/7901241

**Published:** 2020-03-09

**Authors:** Yibekal Manaye, Anemaw Asrat, Endalkachew Worku Mengesha

**Affiliations:** ^1^Department of Biostatistics and Epidemiology, School of Public Health, College of Medicine and Health Sciences, Bahir Dar University, Bahir Dar, Ethiopia; ^2^Department of Reproductive Health and Population Studies, School of Public Health, College of Medicine and Health Sciences, Bahir Dar University, Bahir Dar, Ethiopia

## Abstract

**Methods:**

A retrospective follow-up study was conducted among clients on ART from 2012 to 2017. Data were collected using checklists. The Kaplan-Meier curve was employed to compare survival rates. The Cox proportional hazard model was applied to identify predictors of time to development of anemia.

**Results:**

A total of 490 ART patients were followed. The overall incidence of anemia was 27/100 person-years. The incidence was highest in the second year (18.7/100 PY) of starting ART when compared with the first year (13.8/100 PY) and third year (18.1/100 PY) of ART initiation. The independent predictors show an association for time to development of anemia and were as follows: being female (AHR = 2.94, 95%CI = 2.15–4.0), pulmonary tuberculosis positive (AHR = 2.98, 95%CI = 1.62–5.51), baseline weight < 60 kg (AHR = 1.51, 95%CI = 1.19-1.92), and severe acute malnutrition (AHR = 2.0, 95%CI = 1.39-2.89).

**Conclusion:**

Most of the anemia cases occurred after the first year of ART initiation. Pulmonary tuberculosis, baseline weight, nutritional status, and sex were predictors for anemia. Clients with low baseline weight and abnormal nutritional status need to get close follow-up to prevent the risk of early development of anemia.

## 1. Background

Anemia is one of the world's most widespread health problems especially among HIV-infected individuals. It may result from the indirect effects of HIV infection, such as adverse reactions of medications such as zidovudine, opportunistic infections, neoplasms, and nutritional abnormalities arising from anorexia, malabsorption, or metabolic disorders [1–3].

Use of highly active antiretroviral therapy (HAART) is associated with an increase in hemoglobin concentrations and a decrease in the incidence of anemia. Moreover, there is wide variation in the incidence of anemia among HIV/AIDS patients in all over the world [4].

Anemia is a frequent complication that occurs in 20-80% of HIV-infected persons and is associated with faster disease progression and shorter survival. It is a major public health concern in children and adolescents in the developing countries [4, 5].

It is believed that, in developing countries, survival and risk factors of anemia among patients living with HIV depend on a variety of factors, which may also vary greatly with economic, demographic, behavioral risk, and health factors. Being female, lower CD4 count, higher HIV viral loads, and coinfection with tuberculosis (TB) were strongly associated with moderate and severe anemia [6, 7].

Studies conducted in India, Nigeria, and South Africa indicated that HIV-infected patients with age > 30 years, male gender, hemoglobin level < 11 g/dl, body weight < 45 kg, coinfection with HIV and TB, and CD4 count < 100/*μ*l at baseline had significantly higher risk for anemia [8–10].

In Ethiopia, ART service began in 2003 and free ART was launched in 2005. An estimated number of 738,976 Ethiopians are currently living with HIV, and all of them require ART; however, only 426,000 are currently taking ART [11].

According to the 2016 Ethiopian Demographic and Health Survey (EDHS), the prevalence of HIV among women and men aged 15-49 in Ethiopia is 0.9%; HIV prevalence is higher among women than men (1.2% versus 0.6) [12]. Hence, the aim of this study was to assess time to development of anemia and predictors among HIV/AIDS patients initiating ART.

## 2. Methods

The study was conducted in Bahir Dar city at Felege Hiwot Referral Hospital from June 1 to July 31, 2018, among 490 study participants. Bahir Dar city is found in northwest Ethiopia, and it is 560 km away from Addis Ababa. According to the Bahir Dar city health department office report in 2017, the total population of Bahir Dar city was 348,429 [13]. In the city, there were 2 hospitals and 4 health centers in the governmental sector which provide free ART service for HIV/AIDS patients.

A retrospective follow-up study was conducted in Felege Hiwot Referral Hospital to assess time to development of anemia and predictors among HIV/AIDS patients initiating ART.

All HIV/AIDS patients aged greater than or equal to 15 years who were taking ART at Felege Hiwot Referral Hospital from January 2012 to December 2017 were the study population.

Patients were eligible for inclusion if they were HIV positive, aged 15 years or older, and taking ART and if their follow-up time was fixed. The key exclusion criteria considered if the diagnosis was made outside the health institution or if the patients were transferred in were as follows: women who were pregnant before the time of ART initiation or lactating mothers; HIV patients with incomplete intake form, registration form, and follow-up form; and patients who already had anemia at baseline or before the start of the follow-up period.

The total records in the hospital among eligible study population were 4,000. All the records of study subjects on ART follow-up during the five consecutive years with complete information were used to determine incidence and predictors of anemia among patients on ART. Each study participant was selected by using systematic random sampling method. From one random number in the patient's ART unique identification numbers as a starting point, every eighth record was taken as a study participant.

According to WHO classification, anemic patients were categorized into mild, moderate, and severe anemia: mild anemia (hemoglobin 9.0–10.9 g/dl), moderate anemia (hemoglobin 7.0–8.9 g/dl), and severe anemia (hemoglobin less than 7.0 g/dl).

Data were entered to Epi Info 7 and analyzed by using SPSS version 23. Anemia was confirmed by reviewing the patient card and medical registration in the ART clinic. Finally, the outcome of each subject was dichotomized into censored or event.

Kaplan-Meier method was used to compare survival curves. A range of measures of goodness-of-fit was checked using Schoenfeld's global test. Cox proportional hazard regression was employed to calculate adjusted hazard rate and then determine independent predictors of time to event.

### 2.1. Ethical Consideration

Ethical approval and clearance was given by the Institutional Review Board of Bahir Dar University, College of Medicine and Health Sciences. Permission was also obtained from the concerned bodies of Felege Hiwot Referral Hospital. To maintain confidentiality of people living with HIV/AIDS (PLWHA), health professionals working in the ART clinic were abstracting the data. In addition, no personal identifier was extracted on medical records and the recorded data was not accessed by a third person.

## 3. Results

### 3.1. Sociodemographic Characteristics

A total of 490 HIV/AIDS patients who were taking ART at Felege Hiwot Referral Hospital were included in the study. Among the participants, 306 (62.4%), 296 (60.4%), and 305 (62.2%) were females, orthodox, and living in urban residence, respectively (Table 1).

### 3.2. Baseline Clinical, Laboratory, and ART Information of the Study Subjects

Two hundred eighty-nine (59%) of patients were on WHO stage II at the time of HAART initiation, and WHO stage IV accounts for the lowest frequency among others, that is, 19 (3.9%). The mean ± SD of baseline CD4 count and weight for the study participants were 370 cells/*μ*l ± 235 and 57 ± 11 kg, respectively. 255 (52%) participants used different prophylaxis, among them 140 (28.6%) received cotrimoxazole and 179 (36.5%) of the patients had mild acute malnutrition.

The predominant HAART regimen initially prescribed for 271 (55.3%) patients was a combination of stavudine, lamivudine, and efavirenz (1b = (40) d4t (40)/-3Tc-EFV) (Table 2).

### 3.3. Incidence and Development of Anemia

A total of 490 study participants followed retrospectively for the last five years of whom 288 (58.7%) had developed anemia.

The study subjects had experienced anemia in 12,349 person-months (PM) of observations.

The overall incidence of anemia was 27/100 person-years (PY). The incidence was highest in the second year (18.7/100 PY) of starting ART when compared with the first year (13.8/100 PY) and third year (18.1/100 PY) of ART initiation. The median time to develop anemia was 32 months with IQR (29-34 months).

### 3.4. Predictors for Time to Development of Anemia

**Figure 1:**
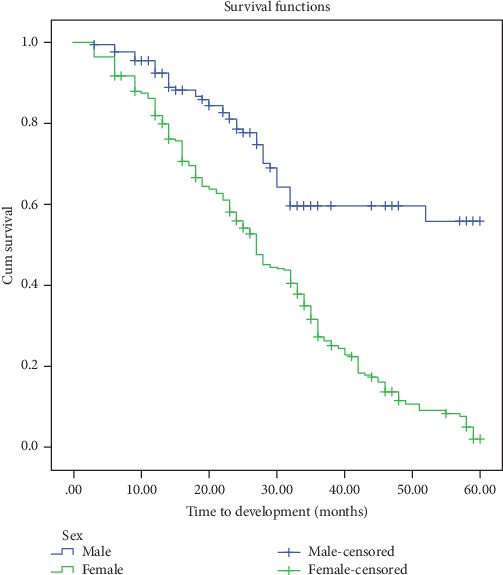
Kaplan-Meier curves for time to development of anemia, in months, among HIV patients on ART, Felege Hiwot Referral Hospital, 2012-2017, classified based on sex.

**Figure 2:**
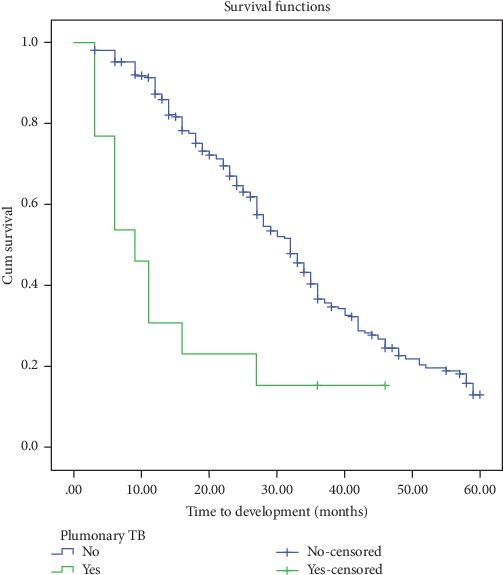
Kaplan-Meier curves for time to development of anemia, in months, among HIV patients on ART, Felege Hiwot Referral Hospital, 2012-2017, classified by past pulmonary tuberculosis exposure.

Based on the multivariable Cox regression analysis, sex, past exposure to pulmonary TB, baseline weight, and nutritional status showed a significant association (Table 3). Patients who are females had higher risk for developing anemia early compared to those who are males (AHR = 2.9, 95% CI: 2.15-4.0). Patients who had baseline weight ≤ 60 kg had higher risk for developing anemia early compared to those > 60 kg (AHR = 1.51 95% CI: 1.19-1.92). Severe acute malnourished patients had higher risk for developing anemia early compared to those patients who had normal nutritional status (AHR = 2.0 95% CI: 1.39-2.89). Patients who had past pulmonary TB had higher risk for developing anemia early compared to those who had no past pulmonary TB (AHR = 2.98, 95% CI: 1.61-5.51) (Figures 1 and 2).

## 4. Discussion

This study determined the incidence rate, time to development of anemia, and its predictors among adult HIV patients taking ART at Felege Hiwot Referral Hospital.

From the total study subjects, 288 (58.8%) of them developed anemia. Of these events, 68 (13.8%) occurred in the first year, 92 (18.7%) in the second year, 89 (18.1%) in the third year, and the remaining in the fourth and fifth years. It was higher than the studies done in multicenters (35.5%) [14], University of Gondar (35%) [4], and southwest Ethiopia (16.2%) [15]. The possible reason for why the variation occurred is because some studies calculated the overall prevalence and others included HAART-naive patients.

The overall incidence of anemia was 27/100 PY with the highest value after 1 year of follow-up. This finding is supported by a study done in the Democratic Republic of the Congo which reported that the incidence of anemia was higher after one year of follow-up [16]. However, a study conducted in Addis Ababa and South Africa revealed that the highest incidence was observed after three years and six months of ART initiation, respectively [10, 17].

The difference between the current study and the previous studies mentioned above might be due to the fact that some of them studied young patients, where such patients need a long period of time to develop anemia. As the age is increased from time to time, the chance of developing anemia early also increased, and some of these studies had a long period of follow-up unlike the current study which is about 5 years.

In this study, female patients had 2.9 (95%CI = 2.15-4.0) times higher risk of anemia than male patients. This was supported by other studies done in Ethiopia [18], South Africa [7], and southern India [16].

This finding was opposite with the study conducted in Zewditu Memorial Hospital, Addis Ababa [1]. In which male patients had 1.55 times higher risk of anemia than patients who are female. The possible reason for the difference might be that in the current study the majority of participants were females, which may be largely attributed to menstrual blood loss. The other reason might be that in the study the majority of participants were old-aged males; evidences showed that early development of anemia would be high as the age increased [17].

Patients who had pulmonary TB in the past had 2.98 (95%CI = 1.6-5.5) times higher risk for developing anemia early than patients who had no previous pulmonary TB. Many studies done in different countries also supported this finding [19]. The study in South Africa showed that patients with pulmonary TB in the past had 1.4 times higher risk for developing anemia early as compared to patients who had no pulmonary TB in the past [10].

A study done in India revealed that patients who had pulmonary TB in the past also confirmed the association but with lower risk than the current study [20]. In the current study area, nutritional abnormality and opportunistic infections are common. So, patients had a low immunity system, and these are aggravated factors for early development of anemia.

Patients who had baseline weight ≤ 60 kg had 1.5 (95%CI = 1.19-1.92) times higher risk for developing anemia early compared to patients who had baseline weight > 60 kg. Other studies done in Southern Ethiopia also supported this finding in which the weight of patients increased by 1 kg and the risk of early development of anemia is decreased by 0.97 [21].

Another study done in Ethiopia, Kombat and Hadiya zones, supported this finding and explained that patients whose weight > 65 kg had a lower risk of developing anemia (0.55) [22].

The possible reason might be that patients who had low body weight are exposed to different types of infections. Due to this reason, they have no resistance for not developing anemia than those patients' weight > 65 kg.

Patients who had severe acute malnutrition had 2.0 (95%CI = 1.39-2.8) times higher risk for developing anemia than patients with moderate and mild malnutrition. And also, patients who had moderate and mild malnutrition had 1.5 and 1.2 times higher risk for developing anemia than patients who are normal, respectively.

The study done in Singapore also supported the current study where patients who had severe acute malnutrition had higher risk for developing anemia than patients with moderate and mild acute malnutrition [23].

Other studies done in Lebanon and Boston showed that patients who had malnutrition had higher risk to develop anemia since malnutrition, deficiencies of micronutrients, and complications with metabolism and body composition are common in HIV/AIDS-infected patients after initiation of ART [24].

### 4.1. Limitation of the Study

Using secondary data in which some important variables were not documented well and many opportunistic infections were presumed diagnoses, future studies will need to be carried out to develop interventions aimed at reducing the incidence of anemia in HIV-infected patients, and additional studies with longer follow-up time should be considered.

Hemoglobin measurement time of some study participants was not constant.

## 5. Conclusion

The overall incidence rate of anemia was high in the follow-up of patients in Felege Hiwot Referral Hospital.

A significant development of anemia was observed after the first year of starting HAART. Sex, past pulmonary tuberculosis exposure, baseline weight, and nutritional status showed a significant association with time to development of anemia.

Effective screening for anemia and giving enough advice for those people living with HIV/AIDS, especially among women and TB-exposed patients, should be designed. A well-integrated nutritional and HIV care system should be demanded to ensure the negative effect of low baseline weight and opportunistic infections like tuberculosis.

## Figures and Tables

**Table 1:** Sociodemographic characteristics of HIV-positive adults at initiation of HAART at Felege Hiwot Referral Hospital (January 2012 to December 2017) (*n* = 490).

Variables	Category	*n* (%)
Sex	Male	184 (37.6)
	Female	306 (62.4)

Age (years)	15–24	114 (23.3)
	25–34	133 (27.1)
	35–44	130 (26.5)
	>45	113 (23.1)

Ethnic group	Amhara	465 (94.9)
	Oromia	8 (1.6)
	Tigray	17 (3.5)

Educational status	No education	151 (30.8)
	Primary school	128 (26.1)
	Secondary school	148 (30.2)
	Higher education	63 (12.9)

Marital status	Single	142 (29.0)
	Married	209 (42.7)
	Divorced	74 (15.1)
	Widowed	33 (6.7)
	Separated	32 (6.5)

Residence	Rural	185 (37.8)
	Urban	305 (62.2)

Occupational status	Farmer	75 (15.3)
	Merchant	132 (26.9)
	Governmental	66 (13.5)
	Daily laborer	50 (10.2)
	Driver	32 (6.5)
	Unemployed	49 (10)
	NGO	66 (13.5)
	Others	20 (4.1)

**Table 2:** Baseline clinical, biological and ART information of HIV-positive adults at initiation of HAART at Felege Hiwot Referral Hospital (January 2012 to December 2017) (*n* = 490).

Variables	Category	*n* (%)
Weight at baseline	≤60	187 (38.2)
	>60	303 (61.8)

BMI	Overweight	60 (12.2)
	Normal	288 (58.8)
	Underweight	142 (29.0)

Functional status at baseline	Working	224 (45.7)
	Ambulatory	202 (41.2)
	Bedridden	64 (13.1)

WHO staging at baseline	Stage 1	94 (19.2)
	Stage 2	289 (59.0)
	Stage 3	88 (18.0)
	Stage 4	19 (3.9)

Past prophylaxis	No	235 (48.0)
	Yes	255 (52.0)

CD4 count at baseline	<200	142 (29.0)
	≥200	348 (71.0)

Baseline ART regimen	1a = (30) d4t(30)/-3Tc-NVP	6 (1.2)
	1a = (40) d4t(40)/-3Tc-NVP	68 (13.9)
	1b = (30) d4t(30)/-3Tc-EFV	101 (20.6)
	1b = (40) d4t(40)/-3Tc-EFV	271 (55.3)
	1c = AZT-3Tc-NVP	27 (5.5)
	1d = AZT-3Tc-EFV	17 (3.5)

Nutritional status	Severe acute malnutrition (BMI < 16 kg/m^2^)	56 (11.4)
	Moderate acute	69 (14.1)
	Mild acute	179 (36.5)
	Normal (BMI 18.5-24.9 kg/m^2^)	186 (38)

BMI: body mass index; d4t: stavudine; 3Tc: lamivudine; NVP: nevirapine; EFV: efavirenz; AZT: zidovudine.

**Table 3:** Predictors of time to development of anemia among adult HIV-positive patients on HAART regimen at Felege Hiwot Referral Hospital (January 2012 to December 2017) (*n* = 490).

Variables	Survival status		CHR (95% CI)	AHR (95% CI)
	Event	Censored		
Sex				
Male	49	135	1	1
Female	239	67	2.97 (2.18-4.05)	2.94 (2.15-4.0)
Past toxoplasmosis				
No	281	191	1	1
Yes	7	11	1.73 (0.82-3.68)	1.68 (0.75-3.74)
Past pulmonary TB				
No	277	200	1	1
Yes	11	2	3.02 (1.65-5.52)	2.98 (1.61-5.51)
Baseline weight				
>60	159	144	1	1
≤60	129	58	1.45 (1.15-1.84)	1.5 (1.19-1.92)
Current diarrhea				
No	228	178	1	1
Yes	60	24	1.29 (0.97-1.72)	1.16 (0.87-1.56)
Nutritional status				
Severe acute malnutrition	46	10	2.37 (1.66-3.40)	2.0 (1.39-2.89)
Moderate acute malnutrition	44	25	1.86 (1.29-2.67)	1.49 (1.03-2.16)
Mild acute malnutrition	109	70	1.41 (1.06-1.87)	1.2 (0.9-1.6)
Normal	89	97	1	1
Current extra pulmonary TB				
No	279	196	1	1
Yes	9	6	1.78 (0.91-3.47)	1.22 (0.62-2.42)
TB prophylaxis				
No	275	185	1.71 (0.98-2.99)	1.64 (0.93-2.91)
Yes	13	17	1	1

## Data Availability

The data used to support the findings of this study are available from the corresponding author upon request.

## Abbreviations

AIDS: Acquired immunodeficiency syndrome
ART: Antiretroviral therapy
AHR: Adjusted hazard ratio
CPT: Cotrimoxazole prophylaxis therapy
HAART: Highly active antiretroviral therapy
OI: Opportunistic infection
PY: Person-year.
